# Trends in delaying and forgoing medical care due to cost and the association with insurance status among US adults with diabetes, 2009–2023

**DOI:** 10.1136/bmjdrc-2025-005446

**Published:** 2025-12-30

**Authors:** Sarah S Casagrande, Jean M Lawrence

**Affiliations:** 1 DLH Holdings Corporation, Atlanta, Georgia, USA; 2 Division of Diabetes Endocrinology and Metabolic Diseases, National Institute of Diabetes and Digestive and Kidney Diseases, Bethesda, Maryland, USA

**Keywords:** Health Care Costs, Epidemiology, Public Health

## Abstract

**Introduction:**

Adults with diabetes require regular medical care which can be costly, but little is known about factors associated with delaying or forgoing medical care due to cost among US adults with diabetes.

**Research design and methods:**

Data were from the 2009–2010, 2014–2015 and 2022–2023 cycles of the cross-sectional National Health Interview Survey and included participants age ≥18 years who self-reported a physician diagnosis of diabetes. Descriptive statistics were used to determine the prevalence and trends in delaying or forgoing medical care by sociodemographic and clinical characteristics and health insurance coverage. Logistic regression models were used to determine the OR for delaying or forgoing medical care associated with insurance status.

**Results:**

Among US adults aged 18–64 years with diabetes, delaying or forgoing medical care due to cost decreased from 18.1% to 10.6% and from 14.6% to 10.2%, respectively, between 2009 and 2023. In 2022–2023, the prevalence of delaying medical care due to cost for adults aged 18–64 years was highest for non-Hispanic black adults (13.3%), those with a high school education or less and poverty income ratio <4.0 (12%–13%). In 2022–2023, uninsured adults ≥18 years were significantly more likely to delay medical care compared with those who were insured (adjusted OR (aOR) =7.5, 4.8–11.8, age 18–64 years (adjusted for sociodemographic and clinical characteristics)). Adults aged 18–64 years with Medicaid were significantly less likely to delay medical care compared with those who had private insurance (aOR=0.2, 0. 1–0.4).

**Conclusions:**

There was a decreasing trend for delaying or forgoing medical care across all subpopulations, but adults with lower education and income and who were uninsured more often reported delays in medical care due to cost. The expansion of Medicaid may have reduced the likelihood of delaying or forgoing medical care due to cost among adults aged 18–64 years with Medicaid coverage.

**WHAT IS ALREADY KNOWN ABOUT THIS TOPIC**

- Diabetes management can be costly. Lack of health insurance has been associated with delays in seeking medical care.

**WHAT THIS STUDY ADDS**

- Delaying or forgoing medical care has decreased since 2009 among adults with diabetes.
- The prevalence of delaying or forgoing medical care was highest for non-Hispanic black adults and adults with less education and income.
- US adults with diabetes who were uninsured were significantly more likely to delay or forgo medical care compared with their insured counterparts.
- US adults aged 18–64 years with Medicaid were significantly less likely to delay or forgo medical care compared with those with private insurance.

**HOW THIS STUDY MIGHT AFFECT RESEARCH, PRACTICE, OR POLICY**

- Health insurance is important for adults with diabetes to maintain regular medical care. In addition, the type of insurance coverage may influence the ability to seek medical care without delay.

## Introduction

 Adults with diabetes require regular medical care, including routine physician visits, laboratory tests, specialty physician visits, and medications, which can be costly.^1^ In the USA, 11.6% of the population has diabetes, which is an estimated 28.4 million people.^2^ Between 2012 and 2022, excess medical costs associated with diabetes increased from US$10 179 to US$12 022 (in 2022 $).^2^ Thus, having health insurance is important for adults with diabetes so that they can access and use the medical care system. Without health insurance, healthcare in the USA is financially burdensome or inaccessible for most people with diabetes who need regular medical care. Health insurance coverage has increased for US adults with diabetes since the enactment of the Affordable Care Act (ACA) in 2010 and the subsequent expansion of Medicaid, a provision of the ACA. Based on data from the National Health Interview Survey (NHIS), the prevalence of health insurance coverage increased from 84% in 2010 to 93% in 2023 among adults with diabetes aged 18–64 years; nearly all adults aged ≥65 years have health insurance due to Medicare coverage. The ACA expanded coverage by providing subsidies for individuals procuring insurance through the Marketplace, which lowered costs for households with incomes between 100% and 400% the federal poverty level; eligibility for health insurance through the Marketplace is only available to individuals who do not have health insurance through their employer, Medicare, Medicaid, or another source that provides qualifying health coverage.^3^ States that expanded Medicaid covered all persons with incomes below 138% the federal poverty line; in 2014, Medicaid had expanded to 26 states and, by 2023, to 40 states.^4^ However, an estimated 1.56 million adults with diabetes still did not have health insurance in 2019.^5^

Previous studies have demonstrated how lack of health insurance is associated with delaying medical care.^6^,^8^ However, there is little research on adults with diabetes and how types of health insurance coverage among the insured are associated with delaying medical care. The USA has a fragmented health insurance system with similarly aged individuals having different coverage (including deductibles, co-payments, and access to specific providers) for healthcare based on their socioeconomic status, employment, and other factors. As such, this study assesses the prevalence and sociodemographic and health insurance-related factors associated with delaying or forgoing medical care due to costs, in a nationally representative sample of US adults with diabetes. In addition, to provide more insight into the mechanisms of the main relationship, modeling techniques are used to account for sociodemographic characteristics and diabetes-related factors that may alter the association between health insurance and delays in medical care.

## Research design and methods

The NHIS is a nationally representative, cross-sectional household survey of the civilian non-institutionalized US population, which has been conducted since 1957 by the National Center for Health Statistics (NCHS). Data from the NHIS are publicly available and de-identified. The NHIS undergoes ethical review by the NCHS Research Ethics Review Board. Detailed information about the survey methods has been described elsewhere.^9^

Study participants included adults aged ≥18 years from the 2009–2010 (n=5466), 2014–2015 (n=7459), and 2022–2023 (n=6172) NHIS survey cycles who answered ‘yes’ to the question: ‘((if female), other than during pregnancy) have you ever been told by a doctor or health professional that you have diabetes or sugar diabetes?’ Diabetes type could not be differentiated. Participants self-reported on sociodemographic characteristics (age, sex, race/ethnicity, education, income, urban/rural residence (2022–2023 only)); health insurance coverage and type (private (with or without a health savings account), Medicaid, Medicare, military), which was not mutually exclusive; as well as clinical characteristics including duration of diabetes (reported as years since first diagnosed with diabetes) and chronic conditions in addition to diabetes (cardiovascular disease, cancer, chronic obstructive pulmonary disease and/or chronic bronchitis or emphysema, depression, work disability (categorized as ≥two chronic conditions)).

### Outcomes

Delaying or forgoing medical care was assessed as part of the healthcare utilization section of the NHIS. Participants were asked if (1) ‘during the past 12 months, have you delayed getting medical care because of the cost?’ and (2) ‘“during the past 12 months, was there any time when you needed medical care, but did not get it because of cost?’. All responses were dichotomous (yes/no). In 2022, a follow-up question on barriers to care was included. Participants were asked, ‘During the past 12 months, did you delay medical care because you had difficulty finding a doctor, clinic, or hospital that would accept your health insurance?’. In the 2022 NHIS, the only year for which these data were available, participants reported on delaying care due to not finding a doctor, clinic, or hospital that accepts their insurance. We included this in our analysis to explore another reason, other than cost, that a person may delay medical care.

### Statistical analysis

In NHIS 2009–2010, 2014–2015, and 2022–2023, descriptive statistics (%, SE) were used to determine prevalence and trends in delaying or forgoing medical care due to cost by sociodemographic characteristics, health insurance coverage, and clinical characteristics. Logistic regression models were used to determine the OR (95% CI) for delaying or forgoing medical care associated with insurance status in 2022–2023, the most current years of data. Models were (1) unadjusted, (2) adjusted for age and sex, (3) additionally adjusted for race/ethnicity, (4) additionally adjusted for education and poverty income ratio, (5) additionally adjusted for urban/rural residence, and (6) additionally adjusted for duration of diabetes and ≥two other chronic conditions.

The NHIS underwent a questionnaire redesign in 2019 that did not affect comparability of variables across our study period (2009–2023). However, to better account for missing income data, the multiple imputation methodology was revised and introduced in the 2019 redesign; prior to 2019, a series of family income bracket questions were used to determine poverty income ratio and data for missing income were imputed.

All statistical analyses used sample weights to account for the complex survey design in accordance with the NHIS analytic guidelines.^9^

## Results

### Sociodemographic factors

**Figure 1:**
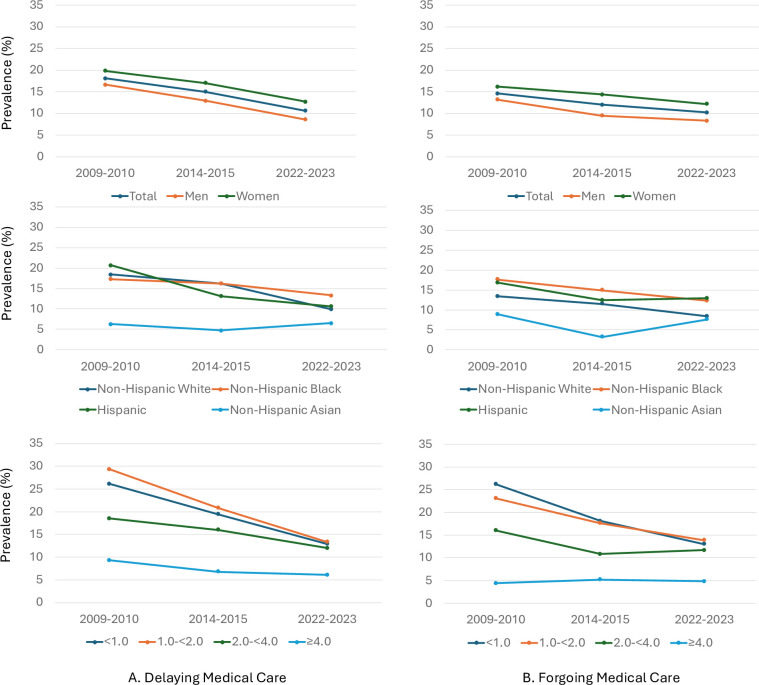
Trends in delaying or forgoing medical care due to cost in the past 12 months among adults with diabetes aged 18–64 years, by sex, race/ethnicity, and poverty income ratio, National Health Interview Survey 2009–2023.

Between 2009–2010 and 2022–2023 among adults aged 18–64 years with diabetes, the prevalence of delaying medical care due to cost decreased for both men and women, and for individuals from all race/ethnicity groups, education levels, and poverty income ratio groups (table 1, figure 1). These trends were similar for forgoing medical care (table 1, figure 1). In 2022–2023, the prevalence of delaying medical care due to cost was higher for women versus men (12.7% vs 8.6%), and highest for non-Hispanic black adults (13.3%), those with a high school education or less (11.9%), those with a poverty income ratio <4.0 (12.0%–13.3%), and those reporting urban versus rural residence (11.1% vs 8.3%).

**Table 1:** Trends in prevalence of delaying or forgoing medical care due to cost in the past 12 months among adults aged ≥18 years with diabetes, by sociodemographic and clinical characteristics and insurance status, NHIS 2009–2023

	Adults with diabetes aged 18–64 years			Adults with diabetes aged ≥65 years		
	2009–2010	2014–2015	2022–2023	2009–2010	2014–2015	2022–2023
	Per cent (SE)					
Delayed medical care due to cost, past 12 months						
Total	18.1 (0.9)	15.0 (0.8)	10.6 (0.7)	5.2 (0.5)	5.0 (0.5)	3.6 (0.4)
Sex						
Men	16.6 (1.2)	12.9 (1.0)	8.6 (0.9)	5.2 (0.8)	4.2 (0.7)	3.5 (0.6)
Women	19.8 (1.2)	17.0 (1.0)	12.7 (1.1)	5.3 (0.7)	5.8 (0.7)	3.7 (0.5)
Race/ethnicity						
Non-Hispanic white	18.4 (1.2)	16.2 (1.1)	9.9 (0.9)	4.2 (0.6)	4.8 (0.7)	3.5 (0.4)
Non-Hispanic black	17.3 (1.5)	16.2 (1.5)	13.3 (2.1)	9.4 (1.7)	6.3 (1.0)	5.3 (1.2)
Hispanic	20.7 (1.9)	13.1 (1.5)	10.6 (1.5)	7.5 (1.3)	6.0 (1.3)	3.9 (1.4)
Mexican American	21.3 (2.2)	14.0 (1.9)	10.9 (1.9)	6.1 (1.2)	6.7 (1.7)	5.9 (2.5)
Non-Hispanic Asian	6.3 (2.1)	4.7 (1.8)	6.5 (2.3)	3.3 (1.7)	2.1 (1.1)	0.9 (0.7)
Education						
Less than high school	21.7 (2.3)	15.8 (1.6)	11.9 (2.0)	7.0 (1.1)	9.0 (1.6)	5.0 (1.3)
High school graduate	17.5 (1.6)	15.3 (1.5)	12.3 (1.4)	5.4 (1.0)	3.1 (0.6)	3.1 (0.6)
Greater than high school	17.1 (1.3)	14.4 (1.0)	9.2 (0.8)	3.7 (0.7)	4.4 (0.6)	3.3 (0.5)
Poverty income ratio						
<1.0	26.1 (2.3)	19.4 (1.6)	12.9 (2.0)	7.8 (1.7)	8.8 (1.7)	3.6 (1.0)
1.0–<2.0	29.3 (2.4)	20.8 (1.9)	13.3 (1.7)	7.8 (1.2)	9.5 (1.5)	6.0 (1.0)
2.0–<4.0	18.5 (1.7)	16.0 (1.5)	12.0 (1.4)	6.1 (1.1)	4.2 (0.9)	3.6 (0.7)
≥4.0	9.3 (1.2)	6.8 (1.1)	6.1 (0.9)	1.4 (0.7)	1.0 (0.5)	1.4 (0.4)
Urban/rural residence						
Urban	NA	NA	11.1 (0.8)	NA	NA	3.8 (0.5)
Rural	NA	NA	8.3 (1.5)	NA	NA	2.8 (0.6)
Insurance status						
Insured	12.3 (0.8)	12.4 (0.7)	8.5 (0.6)	5.1 (0.5)	4.9 (0.5)	3.4 (0.4)
Uninsured	49.5 (2.8)	36.7 (3.3)	36.8 (4.7)	*	*	*
Insurance type						
Private	10.7 (0.9)	11.7 (0.9)	9.6 (0.9)	4.0 (0.7)	2.8 (0.5)	2.3 (0.4)
Medicaid	13.5 (1.6)	11.3 (1.3)	4.9 (0.8)	6.0 (1.6)	6.8 (1.3)	2.5 (0.7)
Medicare	17.8 (2.4)	16.8 (2.0)	12.1 (1.9)	5.1 (0.5)	5.1 (0.5)	3.5 (0.4)
Military	10.2 (2.6)	7.3 (2.1)	5.7 (2.5)	2.9 (1.1)	*	2.0 (0.7)
Duration of diabetes						
<5 years	19.1 (1.6)	15.1 (1.4)	11.7 (1.2)	5.1 (1.1)	4.4 (0.9)	3.3 (0.9)
5–9 years	14.5 (1.3)	15.1 (1.5)	8.8 (1.3)	4.9 (1.2)	7.2 (1.3)	4.3 (1.2)
10–19 years	20.3 (1.8)	16.1 (1.5)	10.8 (1.4)	5.6 (0.9)	4.0 (0.8)	2.8 (0.6)
≥20 years	18.2 (2.4)	13.3 (1.6)	9.9 (1.8)	5.5 (1.1)	5.5 (1.1)	4.3 (0.7)
Chronic conditions, ≥2†	23.7 (1.6)	21.6 (1.4)	13.4 (1.3)	7.2 (0.9)	7.1 (0.9)	5.2 (0.7)
Needed medical care but did not get it due to cost, past 12 months						
Total	14.6 (0.8)	12.0 (0.6)	10.2 (0.7)	3.6 (0.4)	3.7 (0.4)	3.4 (0.4)
Sex						
Men	13.2 (1.1)	9.5 (0.8)	8.3 (0.9)	3.0 (0.5)	2.8 (0.5)	3.6 (0.6)
Women	16.2 (1.1)	14.4 (0.9)	12.2 (1.1)	4.1 (0.6)	4.5 (0.7)	3.2 (0.5)
Race/ethnicity						
Non-Hispanic white	13.4 (1.1)	11.5 (0.9)	8.4 (0.9)	2.3 (0.4)	3.6 (0.6)	3.1 (0.4)
Non-Hispanic black	17.6 (1.7)	14.9 (1.3)	12.3 (2.0)	8.3 (1.4)	4.7 (0.8)	4.3 (0.9)
Hispanic	16.8 (1.7)	12.4 (1.4)	12.9 (1.6)	6.6 (1.5)	4.0 (1.0)	4.7 (1.4)
Mexican American	16.0 (1.6)	12.1 (1.7)	12.8 (2.0)	6.8 (1.9)	4.8 (1.5)	6.6 (2.6)
Non-Hispanic Asian	8.9 (2.9)	3.2 (1.4)	7.6 (2.8)	2.1 (1.2)	1.2 (1.0)	1.4 (0.8)
Education						
Less than high school	19.8 (2.2)	12.7 (1.4)	11.0 (1.9)	4.7 (0.8)	6.9 (1.5)	5.4 (1.3)
High school graduate	14.0 (1.4)	12.3 (1.2)	12.3 (1.4)	2.8 (0.6)	2.6 (0.5)	1.8 (0.4)
Greater than high school	13.1 (1.0)	11.6 (0.9)	8.6 (0.8)	3.4 (0.6)	2.9 (0.4)	3.4 (0.5)
Poverty income ratio						
<1.0	26.2 (2.3)	18.1 (1.5)	13.0 (1.8)	7.4 (1.5)	8.0 (1.5)	5.1 (1.2)
1.0–<2.0	23.1 (2.1)	17.6 (1.5)	13.9 (1.7)	5.7 (1.1)	7.3 (1.4)	5.1 (0.9)
2.0–<4.0	16.0 (1.7)	10.8 (1.2)	11.7 (1.4)	2.9 (0.6)	2.9 (0.7)	3.2 (0.6)
≥4.0	4.4 (0.7)	5.2 (1.1)	4.8 (0.8)	1.2 (0.6)	0.4 (0.3)	1.4 (0.4)
Urban/rural residence						
Urban	NA	NA	10.9 (0.8)	NA	NA	3.5 (0.4)
Rural	NA	NA	6.9 (1.5)	NA	NA	3.1 (0.7)
Insurance status						
Insured	9.0 (0.7)	9.4 (0.6)	8.3 (0.7)	3.4 (0.4)	3.6 (0.4)	3.2 (0.4)
Uninsured	45.2 (2.8)	32.5 (3.1)	35.1 (4.5)	*	*	*
Insurance type						
Private	6.8 (0.7)	7.8 (0.7)	8.7 (0.8)	2.6 (0.5)	1.3 (0.3)	2.2 (0.4)
Medicaid	12.7 (1.6)	11.1 (1.3)	5.9 (0.9)	6.6 (1.4)	6.0 (1.2)	2.7 (0.7)
Medicare	13.1 (1.8)	13.9 (1.6)	12.1 (1.9)	3.2 (0.4)	3.7 (0.4)	3.3 (0.4)
Military	7.4 (2.0)	4.9 (1.7)	6.2 (2.4)	*	*	2.7 (0.8)
Duration of diabetes						
<5 years	16.4 (1.4)	11.7 (1.2)	9.3 (1.2)	2.8 (0.8)	2.8 (0.7)	3.9 (1.0)
5–9 years	11.5 (1.3)	11.6 (1.2)	9.0 (1.3)	3.8 (0.9)	4.1 (1.0)	3.0 (0.8)
10–19 years	14.6 (1.5)	12.6 (1.4)	10.8 (1.3)	4.5 (0.8)	3.1 (0.6)	2.9 (0.6)
≥20 years	14.8 (2.0)	12.0 (1.5)	11.7 (1.9)	3.4 (0.7)	4.6 (1.1)	3.9 (0.7)
Chronic conditions, ≥2†	19.4 (1.5)	18.5 (1.4)	13.5 (1.4)	5.0 (0.7)	4.8 (0.6)	4.6 (0.7)

Information was not available in 2009-2010 or 2014-2015.

* Sample size is too small to provide estimates for uninsured adults aged ≥65 years with diabetes; sample size is too small for some estimates among those with military insurance.

† Chronic conditions (in addition to diabetes) include cardiovascular disease, cancer, chronic obstructive pulmonary disease and/or chronic bronchitis or emphysema, depression, and work disability.

NA, not applicable; NHIS, National Health Interview Survey.

While there was a similar decreasing trend for delaying medical care among adults aged ≥65 years, the magnitude of change in delaying care was smaller (from 5.2% in 2009–2010 to 3.6% in 2022–2023) and there were fewer differences in prevalence by sociodemographic characteristics in 2022–2023 (table 1). Forgoing medical care was stable for adults aged ≥65 years (3.6% in 2009–2010 and 3.4% in 2022–2023) (table 1).

In 2022, among adults with diabetes aged 18–64 years, 5.8% reported delaying medical care due to being unable to find a doctor, clinic, or hospital that accepts their insurance (table 2). The prevalence was higher for women versus men, Hispanic adults vs non-Hispanic white adults, for those with a poverty income ratio <4.0 versus ≥4.0, and for urban versus rural residence.

**Table 2:** Prevalence of delaying medical care due to being unable to find a doctor, clinic, or hospital that accepts their insurance among adults aged 18–64 years, by sociodemographic and clinical characteristics and insurance status, NHIS 2022

	Adults with diabetes age 18–64 years
	2022
	Per cent (SE)
Total	5.8 (0.8)
Sex	
Men	4.1 (0.9)
Women	7.7 (1.2)
Race/ethnicity	
Non-Hispanic white	3.8 (0.8)
Non-Hispanic black	6.8 (1.9)
Hispanic	8.9 (2.1)
Mexican American	8.6 (2.6)
Non-Hispanic Asian	6.0 (3.5)
Education	
Less than high school	6.1 (2.1)
High school graduate	7.9 (1.7)
Greater than high school	4.5 (0.9)
Poverty income ratio	
<1.0	8.6 (2.1)
1.0–<2.0	8.2 (1.9)
2.0–<4.0	7.8 (1.8)
≥4.0	1.0 (0.4)
Urban/rural residence	
Urban	6.6 (0.9)
Rural	1.8 (1.0)
Insurance status	
Insured	5.4 (0.8)
Uninsured	10.5 (4.0)
Insurance type	
Private	3.1 (0.8)
Medicaid	10.5 (2.0)
Medicare	7.1 (2.0)
Military	0.6 (0.6)
Duration of diabetes	
<5 years	6.4 (1.4)
5–9 years	5.0 (1.5)
10–19 years	6.1 (1.6)
≥20 years	5.5 (1.9)
Chronic conditions, ≥2*	8.6 (1.6)

Sample size was too small to provide estimates among adults with diabetes aged ≥65 years.

* Chronic conditions (in addition to diabetes) include cardiovascular disease, cancer, chronic obstructive pulmonary disease and/or chronic bronchitis or emphysema, depression, and work disability.

NHIS, National Health Interview Survey.

### Insurance status

**Figure 2:**
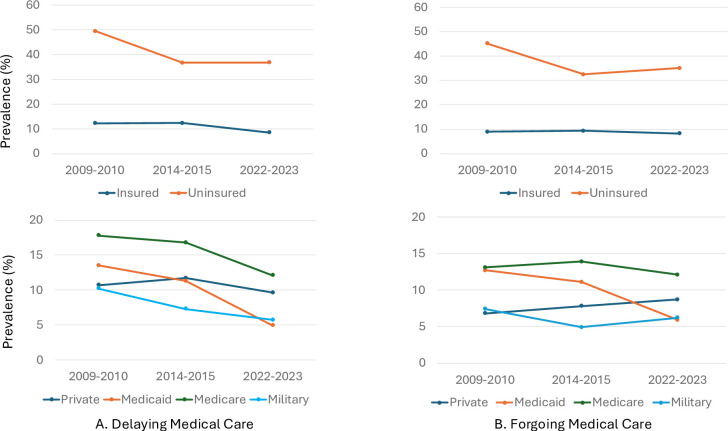
Trends in delaying or forgoing medical care due to cost in the past 12 months among adults with diabetes aged 18–64 years, by insurance status, National Health Interview Survey 2009–2023.

Between 2009–2010 and 2022–2023 among adults aged 18–64 years with diabetes, the prevalence of delaying or forgoing medical care due to cost significantly decreased for those without insurance (49.5% to 36.8% and 45.2% to 35.1%, respectively) (table 1, figure 2). However, the prevalence of delaying or forgoing medical care was much greater for those without insurance versus insurance in 2022–2023 (36.8% vs 8.5% (delaying) and 35.1% vs 8.3% (forgoing), respectively). In 2022–2023, the prevalence of delaying medical care due to cost was lowest for those with Medicaid (4.9%) and highest for those with Medicare (12.1%). This finding was similar for forgoing medical care (Medicaid, 5.9% and Medicare, 12.1%). Among those with private insurance aged 18–64 years, there was no difference in delaying or forgoing medical care for those with or without a health savings account (data not shown).

Delaying or forgoing medical care among insured adults aged ≥65 years was similar between 2009 and 2023 (table 1). Due to almost universal Medicare coverage among adults aged ≥65 years, the sample size was too small to provide reliable estimates for those who were uninsured. In 2022–2023 among adults aged ≥65 years, the prevalence of delaying or forgoing medical care due to cost was low regardless of type of insurance coverage (<4%).

In 2022, adults with diabetes aged 18–64 years with Medicaid and Medicare more often reported delaying care due to being unable to find a doctor, clinic, or hospital that accepts their insurance compared with those with private insurance (10.5% and 7.1% vs 3.1%, respectively) (table 2).

### Clinical characteristics

Between 2009–2010 and 2022–2023 among adults aged 18–64 years, delaying or forgoing medical care due to cost decreased regardless of duration of diabetes (table 1). In 2022–2023 the prevalence of delaying medical care due to cost was similar by duration of diabetes (11.7% with duration <5 years vs 9.9% with duration ≥20 years). Among those with two or more chronic conditions, in addition to diabetes, the prevalence of delaying care decreased from 23.7% in 2009–2010 to 13.4% in 2022–2023. Associations were similar for forgoing medical care due to cost.

Among adults≥65 years, the prevalence of delaying or forgoing medical care was relatively stable between 2009–2020 and 2022–2023 regardless of duration of diabetes (table 1). For those with two or more chronic conditions, the prevalence of delaying medical care decreased slightly from 7.2% in 2009–2010 to 5.2% in 2022–2023.

### Associations with insurance status

In 2022–2023 among adults aged 18–64 years with diabetes, the odds of delaying medical care due to cost were significantly higher for those without insurance compared with those with insurance (adjusted OR (aOR)=7.5, 4.8–11.8) (table 3). In addition, the odds of delaying care were significantly lower for those with Medicaid coverage compared with those with private insurance in unadjusted and adjusted models (aOR=0.2, 0.1–0.4); the association was significantly higher for those with Medicare coverage compared with those with private insurance after adjustment for age, sex, and race/ethnicity (aOR=3.0, 1.6–5.6) but the association was not significant after further adjustment for education and poverty income ratio (aOR=1.9, 1.0–3.6). Similar associations by insurance status and type of insurance were found for forgoing medical care.

**Table 3:** OR (95% CI) for delaying or forgoing medical care in the past 12 months among adults with diabetes aged ≥18 years, by insurance status, NHIS 2022–2023

	Adults with diabetes aged 18–64 years					
	Unadjusted	Adjusted for age, sex	Additionally adjusted for race/ethnicity	Additionally adjusted for education, PIR	Additionally adjusted for urban/rural	Additionally adjusted for duration of diabetes and chronic conditions
Delayed medical care due to cost						
Insured	1.0 (Ref)	1.0 (Ref)	1.0 (Ref)	1.0 (Ref)	1.0 (Ref)	1.0 (Ref)
Uninsured	**6.3 (4.1 to 9.6**)	**6.1 (3.9 to 9.3**)	**6.7 (4.3 to 10.4**)	**6.4 (4.1 to 10.0**)	**6.7 (4.3 to 10.3**)	**7.5 (4.8 to 11.8**)
Insurance type						
Private	1.0 (Ref)	1.0 (Ref)	1.0 (Ref)	1.0 (Ref)	1.0 (Ref)	1.0 (Ref)
Medicaid	**0.5 (0.3 to 0.7**)	**0.4 (0.3 to 0.6**)	**0.4 (0.3 to 0.6**)	**0.3 (0.2 to 0.4**)	**0.3 (0.2 to 0.4**)	**0.2 (0.1 to 0.4**)
Medicare	**2.7 (1.4 to 4.9**)	**2.9 (1.6 to 5.5**)	**3.0 (1.6 to 5.6**)	1.9 (1.0 to 3.6)	2.0 (1.0 to 3.7)	1.6 (0.9 to 3.0)
Military	0.4 (0.1 to 1.2)	0.4 (0.1 to 1.2)	0.4 (0.1 to 1.2)	0.3 (0.1 to 1.0)	0.3 (0.1 to 1.0)	0.3 (0.1 to 0.9)
Needed medical care but did not get it due to cost						
With insurance	1.0 (Ref)	1.0 (Ref)	1.0 (Ref)	1.0 (Ref)	1.0 (Ref)	1.0 (Ref)
Without insurance	**6.0 (4.0 to 9.2**)	**6.0 (3.9 to 9.1**)	**5.7 (3.7 to 8.8**)	**5.8 (3.8 to 8.9**)	**6.1 (3.9 to 9.3**)	**6.8 (4.3 to 10.6**)
Insurance type						
Private	1.0 (Ref)	1.0 (Ref)	1.0 (Ref)	1.0 (Ref)	1.0 (Ref)	1.0 (Ref)
Medicaid	**0.6 (0.4 to 0.9**)	**0.6 (0.4 to 0.9**)	**0.5 (0.4 to 0.8**)	**0.3 (0.2 to 0.5**)	**0.3 (0.2 to 0.5**)	**0.3 (0.2 to 0.5**)
Medicare	**2.6 (1.4 to 4.9**)	**2.7 (1.4 to 5.2**)	**2.7 (1.4 to 5.2**)	1.7 (0.9 to 3.3)	1.7 (0.9 to 3.4)	1.2 (0.6 to 2.4)
Military	0.5 (0.2 to 1.4)	0.5 (0.2 to 1.5)	0.5 (0.2 to 1.5)	0.4 (0.2 to 1.3)	0.4 (0.1 to 1.3)	0.4 (0.1 to 1.1)
Delayed care because cannot find provider that accepts insurance*						
With insurance	1.0 (Ref)	1.0 (Ref)	1.0 (Ref)	1.0 (Ref)	1.0 (Ref)	1.0 (Ref)
Without insurance	2.1 (0.9 to 5.0)	1.9 (0.8 to 4.8)	1.6 (0.6 to 4.1)	1.3 (0.5 to 3.4)	1.4 (0.5 to 3.6)	1.7 (0.7 to 4.5)
Insurance type						
Private	1.0 (Ref)	1.0 (Ref)	1.0 (Ref)	1.0 (Ref)	1.0 (Ref)	1.0 (Ref)
Medicaid	**3.7 (1.9 to 7.1**)	**3.4 (1.8 to 6.5**)	**3.2 (1.6 to 6.1**)	**2.6 (1.2 to 5.6**)	**2.5 (1.1 to 5.5**)	2.1 (0.9 to 4.9)
Medicare	3.2 (1.0 to 10.5)	**3.7 (1.1 to 12.3**)	3.6 (1.0 to 12.2)	3.0 (0.8 to 10.9)	3.1 (0.9 to 11.4)	3.1 (0.8 to 11.4)
Military	†	†	†	†	†	†

	Adults with diabetes aged ≥65 years					
	Unadjusted	Adjusted for age, sex	Additionally adjusted for race/ethnicity	Additionally adjusted for education, PIR	Additionally adjusted for urban/rural	Additionally adjusted for duration of diabetes and chronic conditions
Delayed medical care due to cost						
Insured	1.0 (Ref)	1.0 (Ref)	1.0 (Ref)	1.0 (Ref)	1.0 (Ref)	1.0 (Ref)
Uninsured	**7.6 (2.2 to 26.3**)	**6.7 (2.0 to 22.3**)	**7.4 (2.1 to 26.1**)	**7.2 (2.1 to 24.7**)	**7.1 (2.0 to 24.5**)	**8.9 (2.8 to 28.5**)
Insurance type						
Private	1.0 (Ref)	1.0 (Ref)	1.0 (Ref)	1.0 (Ref)	1.0 (Ref)	1.0 (Ref)
Medicaid	1.2 (0.6 to 2.4)	1.1 (0.5 to 2.4)	1.3 (0.6 to 2.9)	0.7 (0.3 to 1.6)	0.7 (0.3 to 1.5)	0.6 (0.2 to 1.3)
Medicare	**2.3 (1.3 to 4.2**)	**2.4 (1.3 to 4.4**)	**2.5 (1.4 to 4.5**)	**2.1 (1.2 to 3.7**)	**2.1 (1.2 to 3.7**)	**2.0 (1.1 to 3.6**)
Military	†	†	†	†	†	†
Needed medical care but did not get it due to cost						
With insurance	1.0 (Ref)	1.0 (Ref)	1.0 (Ref)	1.0 (Ref)	1.0 (Ref)	1.0 (Ref)
Without insurance	**9.2 (2.8 to 30.3**)	**8.4 (2.7 to 26.2**)	**7.7 (2.3 to 25.5**)	**8.0 (2.4 to 27.1**)	**7.9 (2.3 to 26.8**)	**9.5 (2.9 to 31.1**)
Insurance type						
Private	1.0 (Ref)	1.0 (Ref)	1.0 (Ref)	1.0 (Ref)	1.0 (Ref)	1.0 (Ref)
Medicaid	1.3 (0.6 to 2.6)	1.3 (0.6 to 2.7)	1.3 (0.6 to 2.9)	0.7 (0.3 to 1.4)	0.7 (0.3 to 1.4)	0.6 (0.2 to 1.3)
Medicare	**2.2 (1.2 to 4.1**)	**2.3 (1.3 to 4.3**)	**2.3 (1.3 to 4.2**)	**2.1 (1.2 to 3.8**)	**2.1 (1.2 to 3.8**)	**2.3 (1.3 to 4.1**)
Military	†	†	†	†	†	†

Bold ORs (95% CIs) indicate statistically significant, p<0.05.

* Item only asked in the 2022 NHIS; sample size was too small to assess this item among adults aged ≥65 years.

† Sample size was too small to determine ORs for those with military insurance.

NHIS, National Health Interview Survey; PIR, Poverty Income Ratio.

Among adults aged ≥65 years, delaying care due to cost was significantly higher for those without insurance vs with insurance and those with Medicare versus private insurance (aOR=8.9, 2.8–28.5 and aOR=2.0, 1.1–3.6, respectively) (table 3). These associations were similar for forgoing medical care.

In 2022, adults aged 18–64 years with Medicaid were significantly more likely to delay care because they could not find a doctor, clinic, or hospital that accepts their insurance compared with those with private insurance after adjustment for sociodemographic characteristics (aOR=2.5, 1.1–5.5) but the association became non-significant after further adjustment for duration of diabetes and having ≥ two other chronic conditions (aOR=2.1, 0.9–4.9) (table 3).

## Discussion

In this nationally representative sample of US adults with diabetes, delaying or forgoing medical care due to cost decreased over the past decade; this trend was consistent by sociodemographic characteristics, insurance type, and clinical characteristics. However, being uninsured was significantly associated with delaying or forgoing medical care due to cost compared with those who were insured. Notably, adults aged 18–64 years with Medicaid were significantly less likely to delay or forgo care due to cost compared with those with private insurance. Adults aged 18–64 years with Medicare were also more likely to delay or forgo medical care after adjustment for age, sex, and race/ethnicity, but the association was not significant after further adjustment for education and poverty income ratio. The associations between insurance status and delaying or forgoing medical care were robust and remained significant after adjustment for sociodemographic characteristics.

There are few studies that have assessed the association between health insurance and delays in seeking medical care among adults with diabetes. However, our findings are consistent with studies in other populations. Using NHIS data, it has been reported that among uninsured adults, 68% of cancer survivors and 32% of adults without cancer reported delaying or missing medical care due to cost.^8^ Among adults enrolled in Minnesota’s public health programs, 42% of those who reported financial barriers to medical care delayed medical care in the past year and 23% reported forgoing medical care.^6^ Lastly, findings from another NHIS study showed that adults who were uninsured were six times more likely to delay healthcare due to cost compared with those who were insured after accounting for sociodemographic characteristics and comorbidities.^10^

In 2022–2023 among adults with diabetes aged 18–64 years with private insurance, 9%–10% reported delaying or forgoing medical care due to cost compared with 5%–6% of adults with diabetes insured by Medicaid. Therefore, despite being privately insured, the costs of diabetes care were burdensome enough to delay care. In 2014, a survey conducted in Massachusetts-based emergency departments and outpatient clinics found that privately insured respondents were more likely to delay care due to cost compared with publicly insured respondents.^11^ However, a study conducted in 2010–2015 among non-elderly adults with heart disease showed that publicly insured persons more often delay medical care compared with those with private insurance.^12^ The association between health insurance type and delaying medical care may be nuanced by several factors at both the individual level (eg, sociodemographic characteristics including access to employment-based health insurance, health status, comorbidities, health awareness), the environmental level (eg, number of medical providers, public transportation), and the policy level (eg, state and federal level health insurance related programs).

The decrease in prevalence of delaying or forgoing medical care due to cost was likely due to the implementation of the ACA in 2010 with most provisions mandated by 2014.^13^ First, the ACA expanded coverage by providing subsidies for individuals procuring insurance through the Marketplace, which lowered costs for households with incomes between 100%–400% the federal poverty level.^3^ Second, states that expanded Medicaid covered all persons with incomes below 138% the federal poverty line; in 2014, Medicaid had expanded to 26 states and, by 2023, to 40 states.^4^ Therefore, the expansion of Medicaid likely reduced the prevalence of delaying care due to cost among lower-income persons by raising the income threshold for Medicaid eligibility and reducing the coverage gap. The perceived cost burden of medical care may be less for someone with Medicaid compared with someone with private insurance who is paying premiums, deductibles, which are potentially high, and out-of-pocket co-pays that may limit their healthcare utilization even though they are insured. However, the current study also showed that, in 2022, adults with diabetes and Medicaid were more likely to delay care due to not finding a doctor, clinic, or hospital that accepts their insurance compared with adults with private insurance. This association was significant after adjustment for sociodemographic characteristics but was not significant after further adjustment for duration of diabetes and having at least two other chronic conditions. While we could not explore these findings further, it appears that adults with diabetes and Medicaid had a more difficult time finding physicians compared with their counterparts with private insurance, possibly due to network limitations or administrative barriers. However, when ≥two additional chronic conditions were accounted for in these analyses, adults with Medicaid fared similarly to those with other insurance types in finding a doctor, clinic, or hospital that accepts their insurance. On further exploration, we found that those with Medicaid had more chronic conditions compared with those with private insurance; in 2022–2023 among adults aged 18–64 years, 55.1% with Medicaid had ≥ two chronic conditions versus 20.6% with private insurance (data not shown). Therefore, the extra burden of comorbidities may be associated with the ability to find healthcare that accepts their Medicaid insurance. For example, a person with diabetes and other chronic health conditions on Medicaid may have more challenges in securing healthcare than a counterpart without other chronic health conditions and, thus, be more likely to delay medical care. Nevertheless, a 2017 meta-analysis showed that Medicaid patients had greater difficulty obtaining appointments compared with privately insured patients across a variety of medical facilities; there was no adjustment for individual characteristics, such as comorbidities.^14^ Another study showed that in 2014–2015, the 2 years after implementation of Medicaid expansion, being in an expansion state was associated with more insurance coverage and access to care but also longer wait times for appointments, and thus delays in care, compared with being in a non-expansion state.^15^ As federal-level and state-level health insurance policies may be further modified, future studies can assess how any changes at the policy level impact individuals’ decisions to delay medical care.

NHIS is a cross-sectional survey; thus, causal associations between insurance status and delays in medical care cannot be determined. Limitations of this study were that all data were self-reported, which could have resulted in misclassification of diabetes status, insurance type, or delays in care due to recall bias. While diabetes type could not be determined in NHIS, patients with type 1 and type 2 diabetes both need regular medical care; thus, we would expect any differences in the outcome to be non-differential. The majority of adults with diabetes in the US have type 2 diabetes (90%–95%); therefore, it is possible that the results may not be generalizable to adults with type 1 diabetes. However, this study used a nationally representative sample allowing generalization to the US population with diabetes.

While there has been progress made in reducing delays in care due to cost among adults with diabetes, certain subpopulations more often reported delaying or forgoing medical care due to cost, particularly persons aged 18–64 years with lower education and income and who are uninsured. Over one-third of adults aged 18–64 years who were uninsured reported delaying or forgoing medical care due to cost, which highlights the importance of health insurance among adults with diabetes who need regular medical care. However, the expansion of Medicaid appeared to reduce the likelihood of delaying or forgoing medical care among adults with Medicaid coverage. As private and government-supported insurance has the potential to evolve in the USA, it will be equally important to understand how changes to insurance coverage may impact the ability for patients to manage and control their diabetes and its complications.

## Data Availability

Data are available in a public, open access repository.

## References

[1] Parker ED, Lin J, Mahoney T (2024). Economic Costs of Diabetes in the U.S. in 2022. Diabetes Care.

[2] US Centers for Disease Control and Prevention (2024). National diabetes statistics report.

[3] US Department of Health and Human Services (2022). About the affordable care act. https://www.hhs.gov/healthcare/about-the-aca/index.html.

[4] Centers for Medicare & Medicaid Services (2024). Medicaid eligibility. https://www.medicaid.gov/medicaid/eligibility/index.html.

[5] Casagrande SS, Park J, Herman WH, Lawrence JM, Casagrande SS, Herman WH (2023). Diabetes in America.

[6] Allen EM, Call KT, Beebe TJ (2017). Barriers to Care and Health Care Utilization Among the Publicly Insured. Med Care.

[7] Ratnapradipa KL, Jadhav S, Kabayundo J (2023). Factors associated with delaying medical care: cross-sectional study of Nebraska adults. BMC Health Serv Res.

[8] Sabatino SA, Coates RJ, Uhler RJ (2006). Health insurance coverage and cost barriers to needed medical care among U.S. adult cancer survivors age<65 years. Cancer.

[9] National Center for Health Statistics (2009). NHIS questionnaires, datasets, and documentation. 2009-2023. https://www.cdc.gov/nchs/nhis/documentation/index.html.

[10] Azubuike CD, Alawode OA (2024). Delayed Healthcare Due to Cost Among Adults with Multimorbidity in the United States. Healthcare (Basel).

[11] Al Rowas S, Rothberg MB, Johnson B (2017). The association between insurance type and cost-related delay in care: a survey. Am J Manag Care.

[12] Bernard D, Fang Z (2019). Financial Burdens and Barriers to Care Among Nonelderly Adults With Heart Disease: 2010-2015. J Am Heart Assoc.

[13] Rosenbaum S (2011). The Patient Protection and Affordable Care Act: implications for public health policy and practice. Public Health Rep.

[14] Hsiang WR, Lukasiewicz A, Gentry M (2019). Medicaid Patients Have Greater Difficulty Scheduling Health Care Appointments Compared With Private Insurance Patients: A Meta-Analysis. Inquiry.

[15] Miller S, Wherry LR (2017). Health and Access to Care during the First 2 Years of the ACA Medicaid Expansions. N Engl J Med.

